# Role of Nanotechnology for Design and Development of Cosmeceutical: Application in Makeup and Skin Care

**DOI:** 10.3389/fchem.2019.00739

**Published:** 2019-11-13

**Authors:** Zarith Asyikin Abdul Aziz, Hasmida Mohd-Nasir, Akil Ahmad, Siti Hamidah Mohd. Setapar, Wong Lee Peng, Sing Chuong Chuo, Asma Khatoon, Khalid Umar, Asim Ali Yaqoob, Mohamad Nasir Mohamad Ibrahim

**Affiliations:** ^1^Department of Chemical Process Engineering, Malaysia-Japan International Institute of Technology, Jalan Sultan Yahya Petra, Universiti Teknologi Malaysia, Kuala Lumpur, Malaysia; ^2^Centre of Lipid Engineering and Applied Research (CLEAR), Ibnu Sina Institute for Scientific and Industerial Research, Universiti Teknologi Malaysia, Johor Bahru, Malaysia; ^3^School of Industrial Technology, Universiti Sains Malaysia, Penang, Malaysia; ^4^SHE Empire Sdn. Bhd, Skudai, Malaysia; ^5^School of Chemical Sciences, Universiti Sains Malaysia, Penang, Malaysia

**Keywords:** nanotechnology, emulsion, micelles, cosmetics, skin care

## Abstract

Nanotechnology is an innovative area of science that includes the design, characterization, production, and application of materials, devices and systems by controlling shape and size at the nanometer scale (1–100 nm). Nanotechnology incorporation in cosmetic formulation is considered as the hottest and emerging technology available. Cosmetic manufacturers use nanoscale size ingredients to provide better UV protection, deeper skin penetration, long-lasting effects, increased color, finish quality, and many more. Micellar nanoparticles is one of the latest field applied in cosmetic products that becoming trending and widely commercialized in local and international markets. The ability of nanoemulsion system to form small micellar nanoparticles size with high surface area allowing to effectiveness of bioactive component transport onto the skin. Oil in water nanoemulsion is playing a major role as effective formulation in cosmetics such as make-up remover, facial cleanser, anti-aging lotion, sun-screens, and other water-based cosmetic formulations. The objective of this review is to critically discuss the properties, advantageous, and mechanism of micellar nanoparticles formation in nanoemulsion system. Therefore, present article introduce and discuss the specific benefits of nanoemulsion system in forming micellar nanoparticles for cosmetic formulation which become major factors for further development of micellar-based cosmetic segments.

## Introduction

The Food and Drug Administration (FDA) has defined cosmetics as “particles intended to be applied onto human bodies or any part thereof for cleansing, beautifying, promoting attractiveness, or altering the appearance” (U. S. Food, and Drug Administration, 2018). In general terms, cosmetics is defined as a product that possesses the amplification of skin appearance, beauty and intensify the cleansing (Gautam et al., 2011). Attributable to these definitions, Thornfeldt (2005) was characterized the lists of cosmetics segments and denoted as skin moisturizer, anti-aging, facial makeup, shampoo, toothpaste, deodorant, hair color, and others products that were used for an appearance enhancement (Thornfeldt, 2005).

First time in history, the cosmetics was used in Egypt around 4000 BC. Later, Greeks were used and followed by Romans, Chinese, Japan, and Americans were started to use cosmetics (Gonzalez- Minero and Bravo-Diaz, 2018). In western countries, women are secretly used cosmetics in the late nineteenth century but cosmetics are fully implemented without concealment in the twentieth century (Kaul et al., 2018). In twenty-first century, cosmetics are enormously used and having the high demand in global market which achieved an increment of a year on average (CAGR) by 4.5%, with annual growth rates ranging between 3.0 and 5.5% (Dureja et al., 2005; Saha, 2012). Besides, Asian cosmetics become among the fastest growing demand which reported to achieve an increment of Asia Pacific market value to more than US$70 billion, where Malaysia is becoming a part of it (Hassali et al., 2015).

In cosmetic arena, cosmeceuticals is widely known as a cosmetic product that infused with biologically active ingredient possessing therapeutic benefits on human personal appearance (Dureja et al., 2005; Saha, 2012; Lohani et al., 2014). Cosmeceuticals are considered as niche between pharmaceuticals and cosmetics where the products are enriched with measurable therapeutic efficacies of bioactive components and the formulations are diversified from skin to body to hair in order to use for various treatment such as skin aging, hair damage, skin dryness, dark spots, pigmentation etc. (Lohani et al., 2014; Kaul et al., 2018).

Nanotechnology is known as the most forthcoming technology of twenty-first century and scrutinized as an inventive approach in cosmetic industry. It has been defined as an innovative science that including the design, production, characterizations, and application of science, where the particles can be manipulated in the ranges between 10 and 1,000 nm (Logothethetidis, 2012; Kaul et al., 2018). Nano-cosmeceuticals is elucidated as a cosmetic formulation incorporated with nanotechnology as delivery system to promote an enhancement performance of bioactive components (Hougeir and Kircik, 2012; Kaul et al., 2018). This approach enable to form smaller nanoparticles of cosmetic ingredients which can possesses to active components-readily absorbed onto the skin, repair damage easily and promote better product efficacies (Singh et al., 2013).

Several cosmetics quality and performance have been enhanced by incorporating bioactive components of cosmetic formulation with novel nanocarriers; liposomes (Soni et al., 2016), niosomes (Yeh et al., 2013), solid lipid nanoparticles (Souto and Müller, 2008), nanocapsules (Rosset et al., 2012), micelles (Yukuyama et al., 2016), dendrimers (Mu and Sprando, 2010), and metal nanoparticles (Lu et al., 2015). This approach enable to produce cosmetics with long lasting perfume, better UV protection, and enhanced anti-aging effect. These are owing to smaller nano size of cosmetics' bioactive components after incorporated with nanocarriers, which enhanced their therapeutic effects (Lohani et al., 2014).

Micellar nanoparticles technology is considered as one of the efficient and latest nanotechnology-based cosmeceutical that has been enormously implemented in skin cleanser product segments (Sanjukta et al., 2016; Fukui, 2018; Haziqah et al., 2019; Morganti and Coltelli, 2019). This nanotechnology offers robust and versatile delivery system to incorporate with wide range of lipophilic bioactive components having numerous physicochemical properties that suitable to be incorporated in cosmetics formulation. Being compared with the other nanocarriers such as liposomes and niosomes, micellar nanotechnology promotes smaller nanoparticles, better encapsulation efficiency, and affordable production cost (Sonneville-Aubrun et al., 2004; Lee et al., 2010). Bioderma, L'Oréal, Avenue, Laroche-posay, Garnier, and other international and local brands are claiming their micellar facial cleanser to be the most efficient product due to the cleanser formulation incorporation with micellar nanotechnology. For the successful performance, micellar nanotechnology was used in these brands. There is high possibility to apply this technique for wider cosmetic products segment.

Oil in water emulsion system is one of micellar nanoparticles formation techniques that broadly investigated and reported to be the most suitable system to be implemented in cosmetics formulation. Micelles form via oil in water emulsion require two immiscible liquid containing oil and water phase and surfactant use to stabilize the emulsion. Generally, the mechanism involves the reduction of interfacial tension between oil and aqueous phase by using surfactant, where the surfactant hydrophobic part is attracting toward the oil phase, while hydrophilic part attracts toward aqueous solution. At certain surfactant concentration, known as critical micellization concentration (CMC), the oil phase is entrapped by surfactant hydrophobic segment, while another segment is continuously bonded with entire aqueous medium and form very tiny oil droplets present as micelles (Lee et al., 2010).

This nano-system involves conventional emulsions, microemulsions, and nanoemulsions. The emulsions have been categorized based on their micellar particle size and thermodynamic stability. Due to better properties, nanoemulsion system is becoming the most suitable delivery system attributable to the system capability in forming smaller micelles particle size at lower surfactant concentration (Sonneville-Aubrun et al., 2004). A leading cosmetic company, L'Oreal S. A has patented its micellar-based cosmetic formulations through nanoemulsion system which has various useful application in skin, hair, scalp, mucous membrane and eyes. Besides, a Malaysian cosmetic brand, Naturel Kiss introduces “Micellar Series” product segments which are micellar facial cleanser and mist that infused with various plants' bioactive components incorporated with micellar nanoparticles in nanoemulsion system. Hence, this manuscript is about to review the role of nanoemulsion system in developing micellar nanotechnology-based cosmetics and discuss about the mechanism, advantageous, characterization, and application in makeup and skin care.

## Topical Route of Active Ingredient

Skin is a major human body component as delivery medium of cosmetic active ingredient, which contain multi-layered structure; (1) stratum corneum that composed with dead keratinized cells, (2) epidermis and dermis, and (3) sub-cutaneous tissue. Stratum corneum (SC) is acting as an excellent barrier properties to the skin. Due to keratinized cells of SC containing lipid matrix composed of fatty acids, ceramides, cholesterol, and cholesterol ester which has a cement-like function characteristic (Pawar and Babu, 2014; Yukuyama et al., 2016).

**Figure 1:**
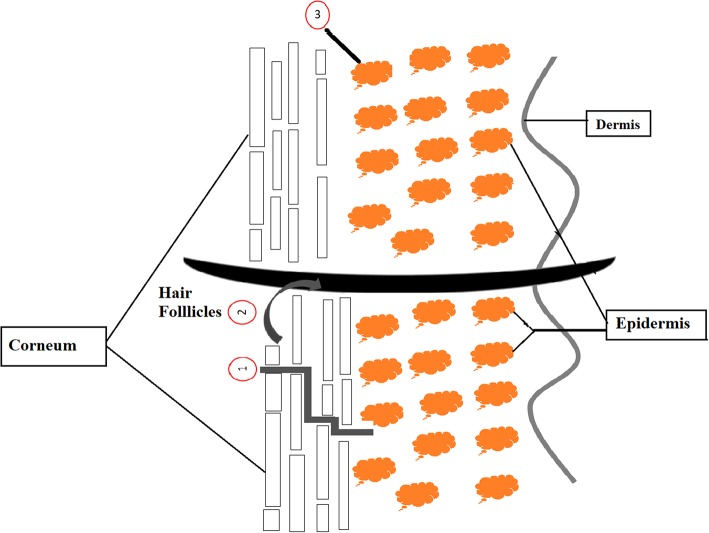
Schematic diagram of three possible way to penetrate applied active ingredient into and through skin (1 = intercellular, 2 = follicular, and 3 = transcellular) (Yukuyama et al., 2016).

Through the topical delivery of active ingredient, there are three possible pathway which are intercellular, hair follicles and transcellular pathways. First, the well-known pathway (intercellular) is taking place where substance diffuses through stratum corneum via the lipid layers surrounded by keratinized cells. Second, delivery path which known as the hair follicles path, functions as a reservoir of active compound topically applied onto skin. This is attributable to the hair follicles part containing a dense network of blood capillaries that serve as supporting penetration medium. Third path is transcellular penetration that involve a direct delivery of active compound through the lipid layers and corneocytes to the living cells (Yukuyama et al., 2016, Mihranyan et al., 2012). Figure 1 shows the illustration of these three pathway types.

In the proportion to the increase in consumer demand of cosmetic products efficacy, there is becoming harder to distinguish the boundary between topical application of cosmetic and pharmaceutical active ingredient (Sonneville-Aubrun et al., 2004). To obtain high efficacy of cosmetic, certain penetration of active compound incorporated with nanotechnology into the skin is required, due to the penetration efficacy dependent on several factors such as molecular size, lipophilicity, and the degree of ionization (Pardeike et al., 2009, Yapar and Inal, 2012). Considering the barrier properties of human skin structure, lipid nanoparticle-based formulation will be one of the most suitable approach for an efficient topical delivery system of active ingredient in cosmetic (Souto and Müller, 2008). Hence, formation of lipid-loaded micellar nanoparticles through nanoemulsion system in the nano scale range is relevant and can be used in cosmetic production.

Historically, micellar nanoparticle technology has been invented by scientist at Novavax in the mid-1990s rolled out the first nano-engineered topical estrogen replacement lotion product, namely Estrasorb^TM^ as an efficient delivery system to enhance the lotion's active ingredient therapeutics. The research has revealed that micellar nanoparticle technology has the ability to allow high concentration of active compound to penetrate the skin effectively and create a drug depot in the stratum corneum and epidermis. This delivery route offers some advantageous in terms of avoiding gastrointestinal tract and hepatic first-pass effect, and this is cosmetically more acceptable toward patients. The development of micellar nanoparticle-based lotion has been done using nanoemulsion system that containing several component which reported to be a delivery system with potentially fast and inexpensive development model in creating more new proprietary formulations (Lee et al., 2010; Rizvi and Saleh, 2018). Due to the successful performance of the first history of micellar nanoparticle application, currently this nanotechnology approach is becoming trending in cosmeceutical industries which being applied enormously among local and international brands. Hence, the next sub-sections of this article contains several review regarding the mechanism, emulsification process techniques and application of micellar nanoparticle formed via nanoemulsion system in commercialized cosmetic products.

## Micellar Nanoparticles in Nanoemulsion System

As mentioned earlier, O/W emulsion is one of the nano-system that can form micellar nanoparticles, where nanoemulsion is becoming the most relevant approach to form micellar-based cosmetic compared to the other emulsions; macro-and microemulsion. Nanoemulsion system is a transparent/translucent solution that containing two immiscible liquids consisting of a fine dispersion of active component structured in nano lipid droplets stabilized by surfactant, which known as micelles or micellar nanoparticles (Lee et al., 2010).

Nanoemulsion (O/W nanoemulsion) has superior property as cosmetic basis compared to the other emulsions in terms of this nano-system ability to form smaller micellar nanoparticle size (10–200 nm) which promote uniform distribution on the skin, enhance active component delivery properties, high stability, larger surface area, pleasant aesthetic character, and skin feel (Sonneville-Aubrun et al., 2004; Yukuyama et al., 2016). The most important role of nanoemulsion based cosmeceutical is the smaller micellar nanoparticle size that possesses high thermodynamically and kinetically stability of cosmetic formulation, against flocculation, sedimentation and Ostwald ripening phenomenon (Anton et al., 2008). The Ostwald ripening, there main mechanism to promote instability of O/W nanoemulsion which is due to a micelles dispersions from small to large droplets through the continuous phase (aqueous solution) (Solans and Solé, 2012). Thus, the small micelles dissolve to form large micelles, affecting the long-term stability of cosmetic formulation which can be a major problem in producing high quality and efficient cosmetic segments (Taylor, 1998).

Through formation of micellar nanoparticles in nanoemulsion, the oil phase play a major role as component that essential to solubilize with lipophilic active component in cosmetic formulation. The amount of oil composition may vary from 2 to 20% w/w based on site administration (Rai et al., 2018). In order to form a highly stable cosmetic formulation, there is only certain types of oil suitable to be implemented. Typically, the oil phase consists of the lipophilic cosmetic's bioactive ingredient such as hydrophobic nutrient, nutraceutical, vitamin, essential oil, color, antimicrobial, or antioxidant agents and carrier oil. The usage of carrier oil is usually to easily facilitate the micellar formation or enhance nanoemulsion system stability (Yukuyama et al., 2016).

Selection of best emulsifier or surfactant in forming micellar nanoparticles are mostly depend on their solubility and emulsification ability (Choudhury et al., 2013). Non-ionic surfactant is the most preferable in cosmetic arena due to their lesser toxicity and irritant compared to ionic (anionic and cationic) surfactant (Azeem et al., 2009).

Furthermore, the use of co-emulsifier or co-surfactant in nanoemulsion system was introduced and claimed to increase the reduction rate of interfacial tension between oil and aqueous phases effectively and thereby increase the entropy of the entire colloidal system (Tirnaksiz et al., 2010). Mainly, glycerin, propylene glycol, propanol, ethanol, Transcutol IP, and ethylene glycol, components among C_3_-C_8_ alcohols have been used as co-surfactant. Its further stabilize the interface and mobility of hydrocarbon chain can be enhanced (Kreilgaard et al., 2000). Table 1 shows the lists of oil components and surfactants used in patented commercialized cosmetic brands (Simonnet et al., 2001, 2002).

**Table 1:** Oil components used in cosmetic preparation.

**No**.	**Oil type**	**Chemical name**
1	Captex 355	Glyceryl tricaorylate/caprate
2	Captex 200	Propylene dicaprylate/dicaprate glycol
3	Captex 8000	Glyceryl tricaprylate (tricaprylin)
4	Witepsol	90:10% w/w C12 Glyceride tri:diesters
5	Myritol 318	C8/C10 triglycerides
6	Isopropyl myristate	Myristic acid isopropyl ester

### Properties and Advantageous of Micellar Nanoparticle Formation in Nanoemulsion

**Figure 2:**
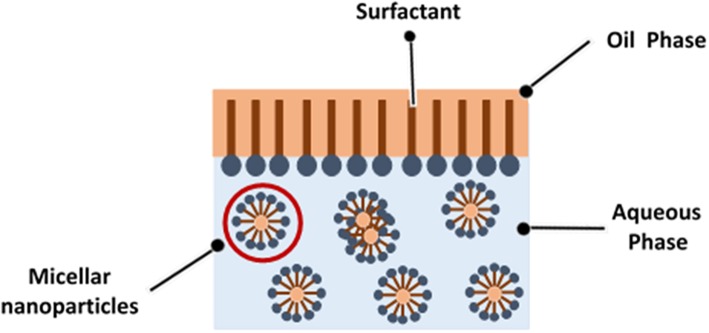
Illustration of micellar nanoparticles formation in nanoemulsion system.

Micelles which formed by surfactant aggregation loaded with bioactive component through nanoemulsion system exhibits various benefits over conventional emulsion such as high stability, enhance absorption rate and solubility of bioactive compound, hence resulted into increase in bioavailability (Zhao et al., 2013; Gorain et al., 2014). Small amount of solvent is used to form micelles in nanoemulsion, therefore it shows high loaded capacity which must be achieved by hydrophilic and hydrophobic segments performance by surfactant, acting on oil-water interface (Sadurní et al., 2005). Figure 2 shows the illustration of micelles formation in nanoemulsion.

The main challenge of nanoemulsion system is thermodynamically unstable which can be overcome by this nano-system ability to form smaller micelles particle size, possesses to more surface charge between micelles droplets and against flocculation, sedimentation, and coalescence help to resist the formation of creaming due to Brownian motion of micelles droplets occurred (Sadurní et al., 2005). Attributable to the possibility of nanoemulsion to overcome any sedimentation or creaming, it offers an enhancement delivery system of bioactive component through crossing the biological membranes barrier by the cellular arrangement reversible alteration and improving the interaction between the cells after solubilisation of lipid barrier interface with the cell wall (Baspinar and Borchert, 2012; Sutradhar and Amin, 2013). This delivery system implemented in delivering bioactive component to the more specific and targeted area, hence possesses to promote high quality and effectiveness of cosmetic formulation. Sub-section below discusses regarding the mechanism involves through the formation of micellar nanoparticles in nanoemulsion system.

### Mechanism of Micellar Nanoparticle Formation

Through the formation of micellar nanoparticles in nanoemulsion system, an emulsification mechanism takes place attributable to interfacial tension reduction between oil and aqueous phase and the formation of charge over the micelles droplets surface, structured by non-ionic, or ionic surfactant (Rai et al., 2018; Abraham et al., 2019). This emulsification mechanism is similar to conventional and microemulsion systems to form micellar nanoparticles.

**Figure 3:**
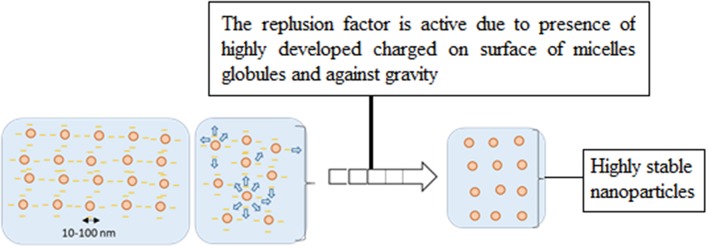
Comparison of droplet size and stability of micelles in (I) conventional emulsion and (II) nanoemulsion (Rai et al., 2018).

Conventional emulsions are in less stable form as compared to nanoemulsions due to large micelles globule size formation. These globules experience less repulsive force between micelles droplets and high gravitational force, and resulted into sedimentation and Ostwald ripening. Micelles containing the oil phase started to separate and moving upward of the system container and become unstable. On the other hand, nanoemulsions form smaller micelles droplets with strongly charge over micelles droplets and promote high repulsive force, resulted into more stable nano-system. Figure 3 show the differenciation of micelles formation between conventional and nanoemulsion systems.

Hydrodynamic breakdown and interfacial modification are the two simultaneous steps involve through nanoemulsion development process. Hydrodynamic breakdown promotes and regulate the dispersed phase to be well-suspended into dispersion medium, while interfacial modification focuses on the surfactants adsorption rate over the interface. These steps are typically occur in a short period, frequently within microsecond during emulsification process (Rai et al., 2018).

Formation of tiny micelles droplets is the important characteristic of nanoemulsion system. In order to form smaller micelles particle size and stable droplets, extensive stress is required to be applied through dispersed phase such as either electric shock, stimuli responsive methods viz. elevated temperature, agitation, or ultrasonic vibrations (Rai et al., 2018). However, the extensive energy used can be avoided due to surfactants function ability to lower the interfacial tension and stabilize micelles droplets. As mentioned, non-ionic surfactants are the most broadly emulsifiers implemented in nanoemulsion system. The one of the best way to choose the best non-ionic surfactant is dependent on its hydrophilic lipophilic balance (HLB) value (Komaiko and McClements, 2015).

Oil-in-water (o/w) nanoemulsion, a system in which the micellar nanoparticle can be formed, requires the HLB of non-ionic surfactant. High HLB value represent the more hydrophilic surfactant, while low HLB surfactant value is considered more hydrophobic surfactant (Alexander et al., 2013). The mechanism of micelles formation through surfactant self aggregation is dependent on the HLB value, where hydrophilic surfactant is more likely to accumulate with aqueous environment and form smaller micelles particle size and stable oil-in-water nanoemulsion (Komaiko and McClements, 2016).

Besides, o/w nanoemulsion stabilization is dependent on the availability of surface charge of surfactant. Non-ionic surfactants may also produced charge on its micelles droplets during dispersed in aqueous phase, where this is attributable to the presence of H_3_O^+^ and OH^−^ ions. The negative charge forms when the nanoemulsion system is in acidic condition (pH 3–6), while positive ion will be observed for natural condition of nanoemulsion system (pH more than 7) (Mitri et al., 2011).

**Figure 4:**
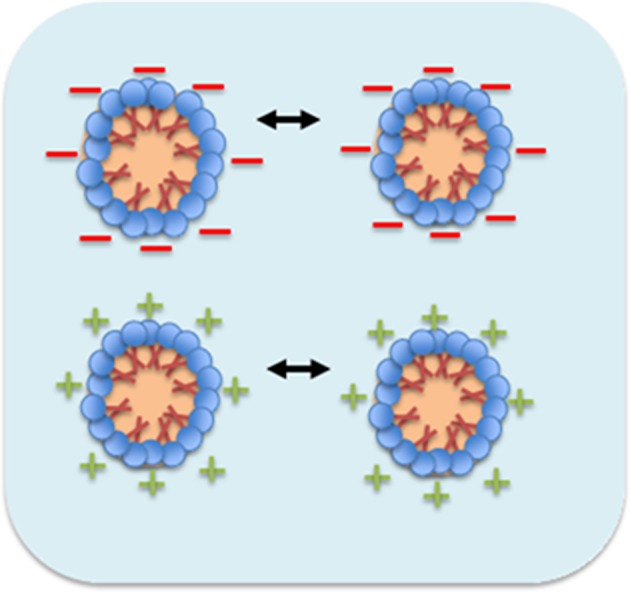
Mechanism of micellar nanoparticles stability in nanoemulsion system (Rai et al., 2018).

The mechanism involves higher degree of surface charge on surfactant aggregation through aqueous medium which possess to keep away between the micelles due to the presence of strong intermolecular repulsive force (Rai et al., 2018). Monosurfactant based nanoemulsion are stabilized by this repulsion force among like charges and promote all the charges of micelles in Brownian motion (Vilasau et al., 2011). Figure 4 shows the micelles droplets in motion approach together with stronger repulsive force among charges, encourage them to be finally move away after an elastic collision through oil in water nanoemulsion system.

## Micellar Nanoparticle Formulation Techniques in Nanoemulsion

There are several emulsification techniques to form micellar nanoparticles in nanoemulsion system which categorized into two process; high energy and low energy techniques. High-energy emulsification technique requires several mechanical forces such as high-pressure homogenizer, ultrasonication, and microfluidization. Besides, the low-energy method involves phase inversion temperature (PIT) and composition (PIC) and spontaneous emulsification methods (Solans et al., 2005). In the cosmetic arena, high energy method is the most reported emulsification technique in forming micellar nanoparticles through O/W nanoemulsion. Meanwhile, recently low energy emulsification technique implementation has grown considerably due to its energy saving approach and being a less damaging process for a labile bioactive constituents (Yukuyama et al., 2016). Therefore, the mechanism involves through the emulsification process using these low and high energy techniques and the findings reported in cosmetic field will be discussed in this paper.

### High-Energy Emulsification Technique: Mechanism

Huge interfacial areas for nanoscale emulsion formation can be formed by implement high-energy technique, since this method process promote the intense mechanical force application (Anton et al., 2008). Through the mechanism of high-energy method, larger droplets are ruptured into smaller droplets due to the mechanical energy, supplies fluid stresses to successful reducing interfacial tension between oil and aqueous phases and resulted into larger total droplets per volume formation (Fryd and Mason, 2012). Generally, two steps are involve to form smaller micelles by using high energy method; (i) final formation of smaller droplets from deformation and distruption of macrosize molecules, and (ii) surfactant adsorption at their interface through aqueous medium to promote steric stabilization (Vladisavljević et al., 2012). Three groups are categorized as typical process of high-energy emulsification method: (i) high-shear stirring using rotor/stator system, (ii) high-pressure homogenization, (iii) ultrasonication (Yukuyama et al., 2016).

In high-shear stirring process, smaller droplets can be formed especially using these well-known rotor/stator type apparatus Ultraturraxpsy® and Omni-mixerpsy. However, this process experienced a difficulty to form average droplet size on a nanoscale by single-regime. In order to overcome this limitation, multiple-regime process was introduced by implement the combination methods. Pre-emulsion can be formed at the beginning using this high-shear stirring, then can be subjected into other process such as high-pressure homogenizer and ultrasonication (Koroleva and Yurtov, 2012).

Through ultrasound or sonication process, smaller micelles droplets are formed by cavitation mechanism where the adequate energy supplied by ultrasonic, offers mechanical depression and compression to collapse the cavitation bubbles and form a large area of the droplets. Several findings revealed that smaller droplets formation is not dependent on the raising of sonication power nor proportional time. Furthermore, surfactant used in the formulation is strongly correlated to formation of tiny micelles droplets. However, ultrasonication process is not practicable for industrial scale which is most appropriate for small batches (Koroleva and Yurtov, 2012; Maali and Mosavian, 2013; Yukuyama et al., 2016). This matter is due to the effective emulsification only occurs in the immediate vicinity of the waveguide radiator, which impacts on the final distribution of the droplet size (Yukuyama et al., 2016).

High-pressure homogenizer process involves the conditions ranging from 10 to 350 MPa which passes through the materials to narrow slot of the homogenizer and experienced with shearing, collision, and cavitation force. The materials that being used must be in medium-to-low viscosity condition, and the final droplets are formed dependent on viscosity of internal phase, where the increase of internal phase viscosity can leads to increase in micelles droplet size. Generally, nanoemulsion is developed in a continous flow with high velocity speed of 100 m/s. The use of high adsorption rate of surfactant is important to avoid any coalescence of newborn droplets. Furthermore, the final size of droplets is based on pressure and temperature of the process condition, in which smaller droplets can be achieved by increasing these conditions (Koroleva and Yurtov, 2012; Maali and Mosavian, 2013).

**Figure 5:**
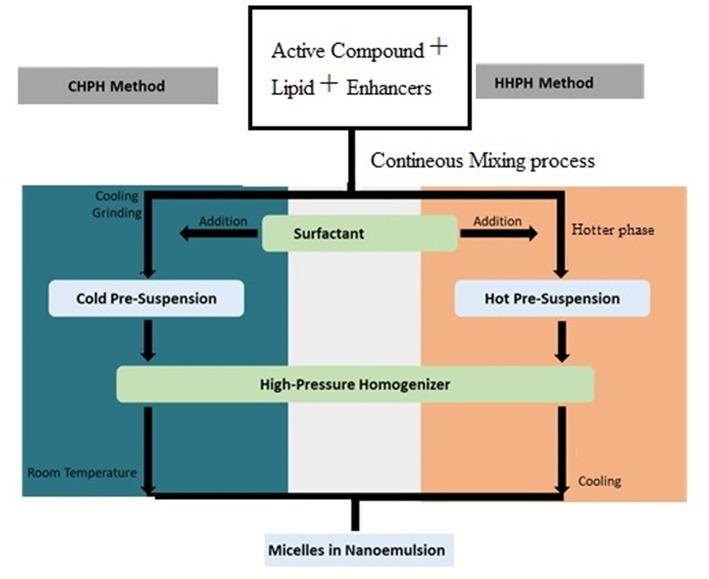
Nanoemulsion system formation through cold (CHPH) and hot (HHPH) high-pressure homogenizer (Adapted from Yukuyama et al., 2016).

This high-pressure homogenizer (HPH) is categorized into two types: hot HPH technique (HHPH) and cold HPH techique (CHPH). These two techniques are differenciate depending on temperature condition of materials or emulsions before passed through the narrow slot of homogenizer (Yukuyama et al., 2016). Extremely temperature-sensitive bioactive component is preferable to be processed using the cold HPH (Müller et al., 2002). In both techniques, the bioactive compound is dissolved, solubilized and dispersed in the melted lipid phase. In hot HPH technique, this mixture is dispersed in a hot surfactant above melting point condition and being stirred by high-speed stirring to form a hot pre-emulsion solution. Besides, in cold HPH technique the mixture of bioactive compound and lipid phase is cooled down, ground and then dispersed into cold surfactant to form a cold pre-suspension phase. Next, for both methods, the pre-emulsion or pre-suspension solution passess through a high-pressure homogenizer at high temperature or room temperature, respectively to obtain smaller micelles in emulsion (Yukuyama et al., 2016). Figure 5 shows the development process of nanoemulsion formation through high-pressure homogenizer involving cold and hot HPH.

Microfluidizer process offers the more advantageous compared to high-pressure homogenizer in terms of short period nanoemulsion formation due to direct emulsification technique by injecting dispersed phase into aqueous phase and form nanoemulsion immediately without any pre-emulsification step. Through this process, the two immiscible liquids, oil and water components flow through microchannels under high pressure of 2000 psi, combined together and processed in inline homogenizer to form nanoemulsion. The uniformity and controlled dispersed phase is dependent on the size of the pores or channels (Koroleva and Yurtov, 2012; Vladisavljević et al., 2012; Maali and Mosavian, 2013).

Besides, the emulsification regime stability is based on the hydrophobicity and hydrophilicity of channel walls. Frequently, in the formation of oil-in-water and water-in-oil nanoemulsions require hydrophilic and hydrophobic surface wall devices, respectively (Koroleva and Yurtov, 2012; Vladisavljević et al., 2012). There are predominantly three types of microfluidizers such as T-junctions, flow-focusing geometries, and co-flowing (Koroleva and Yurtov, 2012; Vladisavljević et al., 2012; Choi et al., 2014). T-junctions is the simplest structure for droplets formation containg inside first developed of microfluidizer. Through microfluidizer channel, there is perpendicular channel passed through by continous and dispersed phases. At the junction, a thin film and shear stress are generated by continous phase during the dispersed phase in contact with the device channel and resulted into generate a droplet by squeezing of dispersed phase (Koroleva and Yurtov, 2012; Vladisavljević et al., 2012; Choi et al., 2014).

Flow-focusing geometries involves the combination of continous and dispersed phases through a small orifice, where at this point dispersed phase is experienced a shear stress generated by continous phase which the narrow size distribution of droplets are able to be formed. In a co-flow microfluidizer, there is a coaxial arrangement where the continous phase is continous flowing. The dispersed phase is injected into capillary tube and both fluids start to flow at low rates which formed a dripping process, where the monodispersed droplets are obtained. Then, either fluid experienced with increased at flow rate, a larger droplet size distribution is formed (Koroleva and Yurtov, 2012; Vladisavljević et al., 2012; Choi et al., 2014).

However, even though microfluidization is the most successful process to form narrower micelles droplets in nanoemulsion system than other emulsification techniques, it has some limitations such as high manufacturing cost, long emulsification period and channels clogged by solid particles, which possesses to re-coalescence phenomenon to happen resulting into larger micelles droplets formation (Fryd and Mason, 2012; Koroleva and Yurtov, 2012).

### High-Energy Technique Application in Cosmetic: Scientific Findings

Several scientific research have been demonstrated the formation of micellar nanoparticles in nanoemulsion system, especially in cosmetic field. Some characterizations and pre-clinical studies have been assessed to validate the efficacy of micellar nanoparticles as cosmeceutical delivery system.

Silva et al. (2013) has formulated a nanoemulsion-based sun protector using ultrasonication technique that containing the micelles of avocado oil droplets as active ingredient. The nanoemulsion system was initially obtained by a composed of avocado oil and different non-ionic surfactant types. A stable nanoemulsion formulation has been successfully developed using surfactant with a longer ethylene oxide chain, and promoted to smaller micelles particle size (6–10 nm). Next, chemical and physical sunscreens component; octyl methoxycinnamate and titanium dioxide, respectively were added to the stable formulation to obtain total SPF 3 of a sun protector. The *in vitro* release evaluation of both sunscreen components demonstrated that the nanoemulsion system was successful possessed a slow and sustained release of octyl methoxycinnamate for a period of 4 h, however the titanium dioxide in the nanoemulsion did not affect any release profile result. This study has concluded that the nanoemulsion system was efficient to enhance UV filter properties of octyl methoxycinnamate, but not for titanium dioxide which showed the diffusion-dependent kinetic of the active ingredient in the formulation (Silva et al., 2013).

In 2011, Kabri et al. were developed a stable nanoemulsion system containing the micelles of omega-3 as cosmetic active compound using high-pressure homogenizer. The nanoemulsion system was formulated by various ingredients such as miglyol, rapeseed and salmon oil as oil phase, soy lecithin, and polyoxyethylene sorbitan monooleate (Tween 80) as surfactant phase, glycerol and deionized water as aqueous phase. Several physicochemical characterizations of the nanoemulsion system have been evaluated and the result revealed that smaller micelles particle size and highly stability of formulation was not only directly dependent on physical parameters of equipment used, but also caused by the properties of surfactant used and composition of oil components. An optimum oil composition was identified; rapeseed oil (56.5%), miglyol (35.5), and salmon oil (8%) with average micelles particle size was 143 nm at polydispersity index of 0.6 (Kabri et al., 2011).

Development and characterizations of micellar nanoparticles of mangosteen extract in nanoemulsion-based cosmetic gel was previously reported. Oil in water nanoemulsion was formulated to enhance the efficacy of mangosteen extract due to its hydrophobic nature which limit the bioactivity effectiveness of the extract as natural anti-bacterial, anti-fungal, anti-oxidant and anti-viral. The nanoemulsion preparation involved virgin coconut oil as carrier oil of mangosteen extract and this mixture was considered as oil phase. Meanwhile, mixed surfactant was used which were the combination of Tween 80 and Span 80 that gave a total HLB value of 12. Span 80 was dissolved in oil phase, while Tween 80 was dissolved in aqueous phase. Then the oil phase was added into aqueous phase while stirring using high-speed homogenizer to form a stable nanoemulsion system (Mulia et al., 2018).

A nanoemulsion-based gel was formulated by mixing the prepared nanoemulsion formulation with the gel base in 1 to 1 volume ratio, under gentle stirring. Next, the formulated nanoemulgel was physicochemically characterized by evaluating its thermodynamic stability, pH value, mangosteen content and *in vitro* skin penetration. The stability test finding showed the nanoemulsion would be stable after being placed in different places and temperature conditions (centrifugation and freeze-thaw cycle) for at least 1 year. Besides, the good stability of nanoemulgel enriched possessed to constant in pH value (5.5–6.4) which the ranges within the human skin pH (4.5–6.5) (Sugumar et al., 2015). The nanoemulgel penetrated the mangosteen-micelles onto skin layer up to μg/cm^2^ within more than 95% of its total mangosteen content. Hence, this research revealed that the nanoemulgel containing mangosteen-micelles is a prospective topical formulation especially for cosmetic.

### Low-Energy Emulsification Technique: Mechanism

Low energy method depends on the inherent physicochemical characteristics of components used such as surfactant, co-surfactant, and excipients compositions. Besides, nanoemulsion is developed by this emulsification technique requires a gentle mixing process and lesser amount of energy compared to high-energy (Anton et al., 2008). Historically, the early stage of micellar nanoparticles formation in nanoemulsion system, high-energy technique was broadly used and investigated such as ultrasonication and high-pressure homogenizer. However recently, the research on low-energy methods for nanoemulsion development is gaining the interest which suitable to be applied for sensitive contituents and energy efficient process for large-scale production [50]. Phase inversion methods; phase inversion composition (PIC), and phase inversion temperature (PIT), and spontaneous emulsification are among the process involve in low-energy method (Yukuyama et al., 2016).

The phase inversion method mechanisms involve the spontaneous change of surfactant curvature radius on interfacial layer based on the manipulated of composition and temperature of the system. At a certain point of composition or temperature, the surfactant adsorbed interfacial layer achieve a zero curvature of the surfactant monolayer where this surfactant zero curvature is structured as lamellar liquid-crystalline phase. With a continously gentle mixing, smaller micelles droplets with low polydispersity index can be obtained. The nanoemulsion with smaller droplets is formed by changing in composition system, is considered as phase inversion composition (PIC) while tiny micelles droplets formation due to tempareture changing of the system, it is called as phase inversion temperature (PIT) method (Yukuyama et al., 2016).

**Figure 6:**
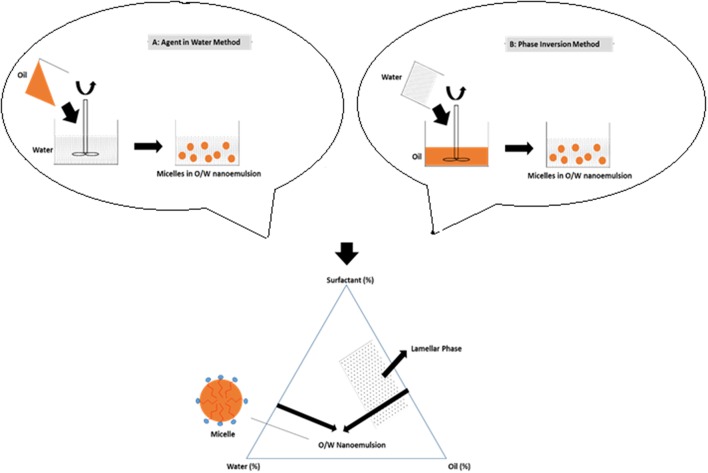
Schematic illustration of micellar nanoparticles formation in O/W nanoemulsion system using phase inversion composition (PIC) (Yukuyama et al., 2016).

Phase inversion composition (PIC) method form the fine micelles droplets dependent on manipulated composition of the system. This method involves the progressive dilution of the oil phase with water or vice versa which form the phase inversion phenomena to obtain nanoemulsion. The hydrophilic/lipophilic balance (HLB) of system is manipulated depending on the increasing or decreasing of surfactant hydration degree according to dilute phase (Yukuyama et al., 2016). The affinity of surfactant toward oil or water phase increases until reaching a zero curvature surfactant phenomenone, where liquid-crystalline lamellar phase or thermodynamically stable microemulsion formed. Then, during the transition composition exceeded, the microemulsion become unstable and breakup to form smaller micelles in unstable but kinetically stable of nanoemulsion system. Through this point, any increasing in composition of the continous dilution phase does not affect the micelles particle size (Anton et al., 2008; Koroleva and Yurtov, 2012; Solans and Solé, 2012; Maali and Mosavian, 2013). Figure 6 shows the illustration of micellar nanoparticles formation in O/W nanoemulsion influenced by different process, but similar composition formula.

Figure 6 shows the method (A) where the oil phase progressively added into water phase, while method (B) the water phase was progressively added into oil phase. Under stirring to form micellar nanoparticles in O/W nanoemulsion system. Through method (B), the final micelles droplets was reported to be smaller compared to method (A), besides only in the method (B) the phase inversion occurred by the presence of liquid lamellar phase.

The phase inversion temperature (PIT) method is depend on temperature and sensitive non-ionic surfactant used in the process system. Frequently, polyethoxylated surfactants are implemented and surfactant HLB composition is fixed and well-balanced while the temperature system is manipulated. The ethoxylated surfactants are enable to be transformed as hydrophilic or hydrophobic dependent on the temperature system. At low temperature, oil-in-water emulsion is obtained due to surfactant area of surfactant being larger at the hydrophilic segments than hydrophobic chains and spontaneously formed hydrophilic curvature of surfactant monolayer (Yukuyama et al., 2016).

As the system temperature increases, attached oxyethylene groups of surfactant become dehydrated and possessed large surface area of surfactant hydrophobic hydrocarbon chains than the hydrophilic segments. Then, hydrophobic or lipophilic curvature of surfactant monolayer is spontaneously formed and resulted into water-in-oil emulsion formation. At an intermediary temperature, the surfactant manifests a similar affinity with water or oil molecules and formed a lamellar liquid-crystalline phase as the interfacial tension and the curvature of surfactant monolayer become zero. At this point, further stirring of the system can develop the tiny micelles droplets, implying that these both phase inversion methods (PIC and PIT) are accessed by same mechanisms (Koroleva and Yurtov, 2012; Solans and Solé, 2012).

Spontaneous emulsification (SE) is one of the low-energy emulsification method that involve various physicochemical mechanisms. True spontaneous emulsification is obtained by emulsifying of two immiscible liquid in contact without any external aid whether in the presence of mechanical or thermal. Solvents can be used to utilize this process in either the presence or the absence of surfactant. Formation of nanoemulsion through SE method with the presence of oil, water and wtaer-miscible solvent with the absence of surfactant is called as ouzo effect. However, the used of solvent is not recommended due to transdermal delivery ingredient safety concerns, therefore the mixture of oil, hydrophilic surfactant, and water components are necessary to generate a nanoemulsion system through spontaneous emulsification method (Saberi et al., 2013; Sugumar et al., 2015; Yukuyama et al., 2016).

Generally, the SE method process involves the slowly titrating or pouring of organic phase containing oil components (including main constituent) and hydrophilic surfactant into aqueous phase (literally water only or mixed with buffer solution) and spontaneously formed the fine micelles droplets due to surfactant diffusion from organic to aqueous phase. This method promotes a simple stirring process to form micelles droplets through nanoemulsion system, instead of expensive high-energy equipments used (Saberi et al., 2013; Sugumar et al., 2015; Yukuyama et al., 2016).

Mainly, the mechanism involves a hydrophilic surfactant movement during titrating organic phase into aqueous phase after mixing which leads to the spontaneous formation of smaller micelles droplets at the oil-water interfacial boundary. Previous investigations have been revealed that the tiny droplets (<100 nm) can be obtained through spontaneous emulsification method by optimizing several factors such as system composition (surfactant and oil type and concentration), and preparation condition (temperature, stirring rate, addition rate). However, the system composition play a major role to be optimized in order to form smaller micelles particle size using spontaneous emulsification technique (Saberi et al., 2013; Sugumar et al., 2015; Yukuyama et al., 2016).

### Low-Energy Technique Application in Cosmetic: Scientific Findings

Several scientific research have been demonstrated the formation of micellar nanoparticles in nanoemulsion system via low-energy emulsification technique, specific to cosmetic field. Some characterizations and pre-clinical studies have been assessed to validate the efficacy of micellar nanoparticles as cosmeceutical delivery system.

A hydroalcoholic extract of *Vellozia squamata*, a medicinal plant cultivated in Brazil was assessed and its micellar formulation was formulated in nanoemulsion system via phase inversion temperature. The formulation was expected to be further used in cosmetic and pharmaceutical fields, due to its specific anti-oxidant properties. Through the phase inversion temperature emulsification technique, the mixture of oil phase and surfactants was heated up to 80 ± 2°C and the water phase at similar temperature was poured onto the oil mixture until the micelles formed.

The findings showed phase inversion temperature was successfully developed stable nanoemulsion system that dispersed with smaller *Vellozia squamata*-micelles particle size (≤ 155 nm) that are adequate for topical application of the extract. Besides, the anti-oxidant activity of pure extract revealed a significant inhibition of DPPH radical and was remained the same for its anti-oxidant properties after being fabricated with micellar nanoparticles. Thus, the study concluded that the nanoemulsion formulation containing dispersions of *Vellozia squamata*-micellar nanoparticles have shown promise as a basis development of phyto-medicines and phyto-cosmeceutical (Quintão et al., 2013).

Barreto et al. (2017) formulated the micellar nanoparticles of polysaccharide-enriched fraction (EF), that isolated from by-product of *A. sisalana* (Barreto et al., 2017). The aim of this study was to develop a micellar-based cosmetic formulation through nanoemulsion system since studies have showed the potential of *A. sisalana*-by product as raw materials of pharmaceutical and cosmetic products development. Besides, a clinical moisturizing efficacy was assessed to determine the potential of micellar nanoparticles in enhancing EF as moisturizing agent. Through the development of formulations, phase inversion composition technique was applied to form the EF-micellar nanoparticles. The technique involved a pseudo-ternary phase diagram consisting of oil phase, aqueous phase (added with EF) and surfactants was designed by varying the components concentrations by 10 (w/w)%.

As the result, it was revealed a stable nanoemulsion formulation containing 40% oil phase, 50% aqueous phase and 10% surfactants were successfully formed with dispersions of smaller EF-micelles particle size that ranges from 83 to 155 nm. The *in vivo* clinical moisturizing analysis demonstrated that 0.5% fraction of total nanoemulsion formulation increased the water content of stratum corneum by 10.13% compared to vehicle and 19.28% compared to baseline values. Besides, the formulation was able to maintained skin barrier function after 5 h of a single application. Hence, the study concluded the EF isolated from *A. sisalana*-by product revealed a promising profile as a moisturizing cosmetic raw material that can be enhanced by fabricated with micellar nanoparticles through nanoemulsion system (Barreto et al., 2017).

Besides in another previous research, Bernardi et al. (2011) developed a nanoemulsion system consisting micellar nanoparticles of rice bran oil, a widely used cosmetic raw material for anti-aging and sunscreen products. The nanoemulsion was formulated by low-energy emulsification technique, phase inversion composition. The total of 42 emulsions were prepared by pseudo ternary phase diagram that involved different concentrations of components, where the oil and aqueous phases were heated separately at 75°C. Through the emulsification process, the water phase was poured into oil phase consisting rice bran oil and surfactants while stirring at 600 rpm, and then the mixture was cooled at room temperature while stirring. This study was chosen a nanoemulsion system with 10% of rice bran oil, 10% surfactants and 80% water as an optimum formulation. This is attributable to this formulation containing smaller rice bran oil-micelles at low surfactant concentration, and since this formulation will be further tested for cosmetic purpose, the micellar formulation that used lowest possible surfactant concentration is preferred.

Through the formulation's efficacy study, an *in vitro* skin irritation analysis showed that the optimum formulation offered a low irritation potential. Meanwhile, the *in vivo* assays demonstrated an improvement on skin's moisture and normal skin pH skin value can be maintained after the formulation application. Thus, it has been concluded the nanoemulsion system ability to be a useful cosmetic delivery system by enriched the rice bran oil with micellar nanoparticles.

### Comparison Between High-Energy and Low-Energy Emulsification Techniques

Basically the high-energy and low-energy techniques have different parameters that playing a major role in developing good characteristics of nanoemulsion system. The stability, micelles particle size and another characterizations of nanoemulsion is affected by quantities of applied energy, the nature of components and amount of surfactant added when facilitated by high-energy emulsification technique. Besides through low-energy method, successful development of nanoemulsion system is depending on the intrinsic physicochemical characteristic and system behavior (Anton et al., 2008; Koroleva and Yurtov, 2012).

The nanoemulsion research progress in developing micellar nanoparticles for cosmetic basis is directly correlation with the diversity of apparatus availability, methodology and focus on recent advance in technology. Attributable to growing interest of nanotechnology implementation in cosmetic arena recently, which target the end customers, it is important to take into consideration regarding the differentiation and impact between laboratory-scale and large-scale production in plant such as cost issue and feasibility (Yukuyama et al., 2016). High-pressure homogenizer and microfluidization are applied to form nanoemulsion system for both laboratory and industrial scale. Meanwhile, ultrasonication process is primarily implemented only for laboratory scale (Maali and Mosavian, 2013).

In terms of energy saving and environmental friendly factors, low-energy emulsification process is more suitable compared to high-energy, since high-energy method requires excessive energy to rupture oil droplets become smaller micellar nanoparticles which resulted into more energy consumption. When emulsification process is occurred via low-energy method, the micellar nanoparticles can be formed in the whole volume of the mixture and spontaneously, then calling-up with ease (Anton et al., 2008). Therefore, low-energy technique is considered as an energy-saving emulsification approach, except for PIT method which heating is required to reach a specific temperature that can successfully form micellar nanoparticles in nanoemulsion system (Koroleva and Yurtov, 2012).

The PIT method is reported to provide a remarkable advantage for industrial production point of view, since this method being a temperature dependent process, it has the flexibility of being repeated for several times by manipulated the temperature to ensure smaller final micelles particle size are formed during production. By comparing with PIC method, where the micellar nanoparticles only can be formed once in the nanoemulsion system. However, in the aspect of stability, through PIT method, it is compulsory to store the final formulation in a temperature far from the PIT temperature to avoid a coalescence phenomenon (Maali and Mosavian, 2013). Other than that, high-energy method requires more investment for industrial-scale production such as equipment and excessive energy consumption, compared to low-energy where the process is based on the chemical energy stored in the nanoemulsion system. However, the high-energy method has an advantage by promoting faster emulsification process compared to low-energy, which benefit for industrial and large-scale production (Yukuyama et al., 2016).

Additionally, composition flexibility factor also need to be taken into consideration among the proposed methods for the micellar nanoparticles formation in nanoemulsion system. The high-energy method is more effective and flexible to introduce various compounds into micelles of the internal phase, since this method is not dependent to the adjustment of temperature changing or adjustment of the interfacial curvature between aqueous and oily phase (Koroleva and Yurtov, 2012). The limitation of high-energy method is in labile drugs and macromolecules such as proteins and nucleic acids that the active components can easily disrupted by intensive shear forces or high temperature (Maali and Mosavian, 2013). Besides, industrial production requires the use of components with low flash points and high flammability as solvents have limitations to be used in cosmetic formulation from a safety point of view. Attributable to this matter, high-pressure homogenizer, microfluidizer and phase inversion methods are more preferred compared to spontaneous emulsification and ultrasonication methods, since these methods do not require any organic solvents (Yukuyama et al., 2016).

Hence, whether it is high-energy or low-energy method, each method promotes its own benefits in terms of many aspect. The formation of smaller micellar nanoparticles size and polydispersity are affected by the method chosen for this preparation. However, the Ostwald ripening that possible to be occurred on the final formulation, is the same, whether the emulsification process involves high-energy or low-energy method. There are two approach recommended to reduce or slow down Ostwald ripening phenomenon occurred in nanoemulsion system; (i) the addition even in a small amount of a substantively lower solubility oil in the bulk phase into the dispersion phase and (ii) the creation of thick steric barrier at the micelles interface by the use of surfactants or emulsifiers (Yukuyama et al., 2016). Therefore, instead of proper selection of emulsification method, the preparation of the component mixture also has a significant influence to a successful characteristics of nanoemulsion system.

## Application of Micellar Nanoparticles in Commercial Cosmetics Products

Micellar nanoparticles-based in cosmetic arena is becoming trending in variety of product segments. Various international and local brands are implementing this nanotechnology as an innovative approach to offer high quality and efficacy of their cosmetic products. Nanoemulsion system is playing a major role to form micellar nanoparticles-enriched with bioactive component, as a potential vehicle for the controlled delivery system of cosmeceutical. This section reviews the commercial cosmetic products that implemented micellar nanoparticles technology.

In New Jersey, the Tri-K Industries has launched a new Nano-based gel for a wide range of skin care products developed by its patent company Kemira. The gel is commercially known as Kemira Nanogel revealed to be a unique nanoemulsion carrier system that has been designed using easy formulation (Chaudhri et al., 2015; Rigano and Lionetti, 2016; Miastkowska et al., 2018). The Nanogel was developed by a simple process to create submicron emulsion containing micellar nanoparticles of bioactive component. The main benefit of the commercialized gel is to minimizing skin water loss, enhanced new skin cell production and easily penetrated of active ingredient. These characteristics have been suggested to be useful for sunscreen, moisturizing and anti-aging of cosmetics segment. Likewise, it also reported that it helps to offer skin care formulations a good skin feel after application (Sharma and Sarangdevot, 2012).

A Malaysian company SHE Empire Sdn. Bhd. has introduced its “Micellar Series” launched by a brand, Naturel Kiss that implemented micellar nanoparticles technology-based cosmetic segments. There are two products that being commercialized; micellar-based facial cleanser and mist. These products are basis with dispersions of micellar nanoparticles of variety bioactive components such as rose, peppermint, lemon, and rosehip essential oils that being developed via nanoemulsion system. Through the commercialization marketing from the company, the micellar-based cleanser offers an efficient process of facial removing make up and dirt, with an infusion of bioactive components-micelles that possesses various skin benefits after cleanse. Besides, the commercialized micellar-based facial mist is specializing for reducing pigmentation, acne, scars and anti-aging benefits. Naturel Kiss is knowing as a leading brand that applied micellar nanoparticles technology in many cosmetic products that being developed by research and development approach. Besides, collaboration between the company's researchers with worldwide scientific committees, several scientific journal have been published regarding successful findings of micellar nanoparticles technology as cosmeceutical delivery system (Aziz et al., 2016, 2017).

A worldwide leading cosmetic company, L'Oreal S. A has two micellar nanoparticles-based cosmetics product which produced nanoemulsion system. Firstly, the invention was successfully developed a transparent and stable nanoemulsion with dispersions of oils-micelles having average particle size of <100 nm. The nanoemulsion system consists of oily phase includes at least one oil having molecular weight more than 400, dispersed through aqueous phase. Meanwhile, the surfactant that being used was at least one anionic surfactant selected from the group including phosphoric acid fatty esters and oxyethylenated derivetives. Through the formulation, weight ratio of the oily phase to the surfactant ranges from 2 to 10%. The good transparency and cosmetic properties of the formulation was reported to be retained eventhough containing with large amount of oil. Besides, the inventors concluded that the micellar nanoparticles-based cosmetic formulation is useful in application of skin, hair, scalp, mucous membrane and eyes (Simonnet et al., 2001).

The second implementation of nanoemulsion system in micellar-based cosmetic formulation patented by L'Oreal was developed the dispersions of micellar nanoparticles loaded with oil components which facilitated using a surfactant comprised of polyethylene oxide and polypropylene block co-polymer. The micellar-based cosmetics that have been formulated are fluid make-up remover, make-up removing gel and eye lotion. These cosmetic segments are dispersed with micellar nanoparticles of oils having molecular weight more than 400 and average particle diameter <100 nm (Simonnet et al., 2002).

## Conclusion

Nanoemulsion has recently achieving a huge demand to form micellar nanoparticles-based cosmetics beause this is due to nano-system potential and efficient delivery system of bioactive components. Small size of micellar nanoparticles can be successfully prepared through nanoemulsion system which promotes higher penetration rate of active component onto skin layers. Highly stable properties of nanoemulsion system posesses to offer no sedimentation, creaming, and sticky feeling after application of cosmetics. In this review, authors discussed the mechanism involves toward the formation of micellar nanoparticles in nanoemulsion system. Small micellar nanoparticles size can be successfully formed by proper hydrophilic-lipophilic balance (HLB) value of surfactant and higher degree of surface charge among surfactant aggregations. Besides, high-energy and low-energy emulsification techniques have been applied to form micellar nanoparticles in nanoemulsion system. This manuscript has reviewed the scientific findings regarding formation of micellar nanoparticles-based in cosmetic formulations using different emulsification technique. Evidently, the application of micellar nanoparticles through nanoemulsion system in commercialized products have also been reviewed. The reviews included Malaysian local brand, Naturel Kiss that launched its “Micellar Series” involving micellar-based facial cleanser and mist that infused with various local plants bioactive components. Additionally, two patent have also been reviewed where the leading cosmetic company L'Oreal S. A has utilized nanoemulsion system to form the micellar nanoparticles-based cosmetic segments. Hence, this review mainly focused on mechanism of smaller micellar nanoparticles formation and its application in commercialized cosmetic products. It also demonstrates that nanotechnology approach has huge potentials to be further implemented in wide range of cosmetic segments. The successful delivery system of nanoemulsion found in cosmetics, can be recommend to be implemented in another field such as food and pharmaceutical.

## Author Contributions

ZA, HM-N, SC, and AK design and written the first draft of review. AA, SM, and WP was responsible for drafting and critically revised the manuscript. MM, AY, and KU participated in technical check and revision of manuscript and draw the figures. All authors discussed the results and commented on the manuscript.

### Conflict of Interest

SM is a Managing Director of SHE Empire Sdn. Bhd, a Spin-off company of Universiti Teknologi Malaysia. The remaining authors declare that the research was conducted in the absence of any commercial or financial relationships that could be construed as a potential conflict of interest.
